# Anabolic steroids abuse and male infertility

**DOI:** 10.1186/s12610-016-0029-4

**Published:** 2016-02-06

**Authors:** Rabih El Osta, Thierry Almont, Catherine Diligent, Nicolas Hubert, Pascal Eschwège, Jacques Hubert

**Affiliations:** Urology Department of Brabois University Hospital, CHU de Nancy, Allée du Morvan, 54511 Vandœuvre les Nancy, France; EA3694 Human Fertility Research Group, CHU Paule de Viguier, TSA 70034, 31059 Toulouse cedex 9, France; Laboratory of reproductive biology, CHU de Nancy, 10 rue du Dr Heydenreich, 54042 Nancy, France

**Keywords:** Anabolic androgenic steroids, Male infertility, Hypogonadism, Anabolisants androgènes stéroïdiens, Infertilité masculine, Hypogonadisme

## Abstract

For several decades, testosterone and its synthetic derivatives have been used with anabolic and androgenic purposes. These substances were first restricted to professional bodybuilders, but become more and more popular among recreational athletes. Up to date, 3,000,000 anabolic-androgenic steroids (AAS) users have been reported in the United States with an increasing prevalence, making AAS consumption a major public health growing concern. Infertility is defined by the WHO as the failure to achieve a clinical pregnancy after 12 months or more of regular unprotected sexual intercourse and a male factor is present in up to 50 % of all infertile couples. Several conditions may be related to male infertility.

Substance abuse, including AAS, is commonly associated to transient or persistent impairment on male reproductive function, through different pathways. Herein, a brief overview on AAS is offered. Steroids biochemistry, patterns of use, physiological and clinical issues are enlightened. A further review about fertility outcomes among male AAS abusers is also presented, including the classic reports on transient anabolic steroid-induced hypogonadism (ASIH), and the more recent experimental reports on structural and genetic sperm damage.

## Resume

Depuis plusieurs décennies, la testostérone et ses dérivés synthétiques ont été utilisés à des fins anaboliques et androgéniques. Initialement réservées aux culturistes professionnels, ces substances ont été progressivement utilisées par les athlètes et les pratiquants de la musculation.

Actuellement, pas moins de 3 millions d’utilisateurs de stéroïdes anabolisants ont été signalés aux États-Unis, et la prévalence croissante de cette utilisation fait de ce phénomène un sujet de préoccupation majeur.

L’infertilité est définie comme l’incapacité à obtenir une grossesse réussie après 12 mois ou plus de rapports sexuels réguliers non protégés, le facteur masculin étant impliqué dans 30 à 50 % des cas parmi tous les couples infertiles.

L’abus de substances, y compris les anabolisants, est souvent associé à une altération transitoire ou persistante sur la fonction reproductive mâle par différentes voies.

Un aperçu des produits anabolisants couramment utilisés ainsi que leurs modes d'utilisation et mécanismes d’action sont présentés. Les implications sur la fertilité sont détaillées. Outre l’inhibition axiale transitoire, les dommages structurels et génétiques des spermatozoïdes, connus à ce jour, sont décrits. Enfin, la spécificité et les modalités de la prise en charge thérapeutique de cette catégorie de patients infertiles sont évoquées.

## Introduction

For several decades, testosterone and its synthetic derivatives have been used with anabolic and androgenic purposes. Initially, these substances were restricted to professional bodybuilders, becoming gradually more popular among recreational power athletes.

Up to date, 3,000,000 Anabolic-Androgenic Steroids (AAS) users have been reported in the United States [1, 2], and considering its increasing prevalence, AAS consumption has become an issue of major concern. When combined with a proper diet and an intense training program, anabolic steroids are able to increase strength and muscle mass in some people. They have many side effects which can be permanent or potentially fatal. Most side effects are mild and reversible like the alteration of the male reproductive system, discussed in this article.

The abuse of anabolic steroids, is often associated with transient or persistent alterations of male reproductive function by different routes.

## Background

### Epidemiology

Since testosterone isolation and characterization in 1935, many derivatives have been synthetized, which properties differ from those of testosterone. These derivatives are called anabolic-androgenic steroids (AAS), or more commonly, anabolic steroids. Initially, these substances were restrictly used by professional athletes and bodybuilders. Nowadays, non-professional and recreational use become more and more popular.

Recent international studies reported an overall lifetime prevalence of AAS use for men of 3–4 % [3] and of 1.6 % for women [4]. AAS use among male gym attendees is estimated to be as high as 15–25 %, depending on the country and with an increasing prevalence [5–7]. Actually, 3,000,000 AAS users have been reported in the United States [1]. By contrast, AAS use is rare in Eastern Asia [2]. Nevertherless, rates of AAS use are also high in Scandinavia, Brazil, British Commonwealth countries, and in Europe [2]. This increasing prevalence of AAS consumption in become a major public health issue in these countries.

Perry et al. studied the steroid regimen (called “cycle”) of 207 AAS consumers. The authors reported an average consumption of 3.1 product per person and per cycle during the 5 to 10 weeks cycles, with doses often being 5 to 29 times the physiological replacement doses [8]. Dependence to AAS was reported by 33 % of respondents. These results suggest that the use of AAS among weightlifters and bodybuilders, usually involving different types of steroids and several additional dietary agents, can lead to adverse reactions, polypharmacy, high doses consumption and addiction for at least 1/3 of them.

### Law

According to Article L232-9 of the Code of Sport in France [9], it is forbidden for any athlete to hold or use, without duly justified medical reason, dopants or restricted products at sporting competitions or events. Concerning Prohibition of this practice, the World Anti-Doping Code [10] has proven to be a very powerful and effective tool in the harmonization of anti-doping efforts worldwide, but not enough to fight against this illegal phenomenon.

## Physiopathology

Testosterone is the most important androgen in the human body. Effects of androgens are most evident during puberty, when they induce deep physiological changes in the male body: development of secondary male characteristics, hair growth pattern, sebaceous gland activity, sperm maturation and libido. Testosterone has several possible metabolic fates [11].

First, it binds to the androgen receptor (AR) in target tissues to exert its effects. Second, it is reduced to 5 *α*-dihydrotestosterone (5DHT), which also acts on the AR. Following a different path, testosterone may be aromatized to estradiol to exert estrogenic effects, typically water retention, breast tissue growth and an increase in body fat deposition.

### Anabolism and androgenism

Anabolism is defined as any state in which nitrogen is differentially retained in lean body mass through the stimulation of protein synthesis and/or a reduction in protein breakdown [12]. It includes growth promotion, protein and collagen synthesis and an increase in muscle size and bone metabolism.

Androgenism is defined as physiological changes in the male body, including the onset of secondary male characteristics, hair growth pattern, sebaceous gland activity, maturation of sperm and libido.

Characteristically, more anabolic steroids present weaker AR bindings, and more androgenic steroids strongly bind the AR, exerting a more potent effect.

### Anabolic steroids: beyond testosterone

Structural changes have been made to the testosterone molecule in order to maximize the anabolic effects and minimize the androgenic ones. However, all AASs are virilizing if administered for long time enough, at high enough dosages [13]. Traditionally, AASs are classified into two categories according to the route of administration and their carrier solvent [2, 14, 15]:

- *Oral AAS or 17 α-esterified steroids:*
  17 *α*-alkylated AAS group originate from the substitution of the 17 *α*hydrogen on the steroid nucleus for a methyl or ethyl group. Alkyl substitution prevents deactivation of the steroid by hepatic first-pass metabolism (necessitating hepatic monitoring), which promotes oral activity. They usually have short half-lives, making daily doses necessary to maintain appropriate blood levels. This class includes the very common stanozolol and oxandrolone, as well as methyltestosterone and others.
- *Parenteral AAS or 17 β-esterified steroids:*
  Usually, the 17 *β*-hydroxyl group is oesterified with an acid moiety to prevent rapid release from the oily vehicle. Roughly, the longer the chain of the acid moiety is, the more slowly the preparation is released into the blood stream. Once in the circulation, hydrolysis rapidly occurs yielding the active compound. They usually have a longer half-life and a slower absorption rate, bringing much less hepatic stress than the orally taken steroids. Pain at injection sites is common, because of the oily base.

There are four basic active compounds:

1. *testosterone*, bound to esters such as undecanoate, cypionate, propionate and others;
2. *19-nortestosterone* (or nandrolone), also bound to different esters. Nandrolone is extremely popular, owing to its high anabolic: androgenic ratio. In contrast to testosterone, nandrolone is converted to a less potent metabolite after 5 *α*-reduction. This, in addition to nandrolone’s lesser affinity to AR, explains the higher myotrophic: androgenic ratio;
3. *boldenone*, bound to ester undecylenate;
4. *trenbolone*, bound to ester acetate.

Anabolic-androgenic steroids may also be classified according to their main effects as follows [2, 14, 15]:

- The *“Testosterone-like” effect:* The testosterone-like effect is very potent, and allows great muscle strength gains. These AASs usually show an anabolic/androgenic ratio close to 1:1, similar to testosterone itself. The high aromatization rates are also comparable with those of testosterone. They include all testosterone esters, methyltestosterone and others.
- The *“Dihydrotestosterone (DHT)-like” effect:* The DHT-like effect is potent but highly androgenic. As these AASs resemble a 5DHT molecule, they cannot be aromatized to estrogen and they also have a low water and salt retention. These AASs include stanozolol and oxandrolone.
- The *“Nandrolone-like” effect:* The nandrolone-like effect is the least potent of all, with the highest anabolic/androgenic ratio. The AASs in this group have some progesterone-like activity, inhibiting the hypothalamic axis. These AASs are the most frequently used drugs in the clinical setting, when anabolic effects are desired (they reverse catabolic states, such as AIDS-associated cachexia, severe burns, and chronic obstructive pulmonary disease). They include the nandrolone esters and trembolone.

### Modes of use

Different oral and injectable compounds are generally combined (‘stacked’), creating large dose regimens, usually self-administered during 4–12 weeks long-lasting periods (‘cycles’) [16]. ‘Stacking’ is based on the idea that smaller dosages of multiple drugs might reduce the chance of complications than larger dosages of a single drug. This may also facilitate the administration of multiple AASs (necessary to achieve supraphysiological doses) for longer periods, and so minimizing the plateauing effect.

The aim of ‘stacking’ is to rationally combine different characteristics, avoiding overlap of benefits or side effects. ‘Mass-building stacks’ consist of the combination of testosterone and nandrolone (or similar drugs), used to maximize muscular and strength gains. ‘Cutting stacks’ consist of combinations containing potent androgens, which are preferred for dieting and body definition, because of their lack of estrogenic activity (less water, salt and fat retention). Heavy users may combine a ‘mass-building cycle’, then ‘cutting cycle’, to finish by a ‘post-cycle therapy’ including anti-estrogens or human Chorionic Gonadotropin (hCG), to try to restart androgen production by the testicles. The Table 1 recapitulates the different products used, their commercial name, their prices and active agent.

**Table 1:** Common oral and injection steroids available through the Internet

Type of product	Administration mode	Trade name	Composition	Active agent (Formula)
Anabolic Androgenic Steroids (AAS)	Injection	Boldenone 300	Boldenone Undeclynate	Boldenone (C_19_H_26_O_2_)
		Masteron 100	Drostanolone propionate	Drostanolone (C_23_H_36_O_3_)
		Winstrol 50 mg	Stanozolol	Stanozolol (C_21_H_32_N_2_O)
		Trenbolone A100	Trenbolone Acetate	Tremblone (C_18_H_22_O_2_)
		Trenbolone E200	Trenbolone Enanthate	
		Tri-Trenbo	Mixture of trenbolones: Trenbolone Enanthate Trenbolone Acetate Trenbolone Hexahydro-benzylcarbonate	
		MixDeca	Mixture of nandrolone: Nandrolone propionate Nandrone phenylpropionate Nandrolone decanoate Nandrolone laurat	Nandrolone (C_18_H_26_O_2_)
		Deca Rapide	Nandrolone Phenypropionate	
		Deca durabolin 300	Nandrolone decanoate	
		Sustanon 300	Mixture of testosterones: Testosterone propionate Testosterone phenylpropionate Testosterone isocaproate Testosterone decanoate	Testosterone (C_19_H_28_O_2_)
		Testosterone P100	Testosterone phenypropionate	
		Testosterone C250	Testosterone Cypionate	
		Testosterone E300	Testosterone Enanthate	
		PharmaMix-1	Mixture of: Testosterone Cypionate Boldenone Undecylenate Testosterone Phenylpropionate	-
		PharmaMix-2	Mixture of: Trenbolone Acetate Drostanolone Propionate Testosterone Phenylpropionate	-
		PharmaMix-3	Mixture of: Trenbolone Enanthate Nandrolone Decanoate Testosterone Enanthate	-
		Primobolan 100	Metenolone enanthate	Metenolone (C_27_H_42_O_3_)
	Oral	Anavar 10 mg	Oxandrolone	Oxandrolone (C_27_H_42_O_3_)
		Dianabol 10 mg	Methandienone	Methandienone (C_20_H_28_O_2_)
		Winstrol tabs 10 mg	Stanozolol	Stanozolol (C_21_H_32_N_2_O)
		Turanabol 10 mg	Turanabol	Turanabol (C_20_H_27_ClO_2_)
		Anapolon 50 mg	Oxymetholone	Oxymetholone (C_21_H_32_O_3_)
Burners		Clenbuterol – Meditech	Clenbuterol hydrochlorid	Clenbuterol (C_1_H_18_Cl_2_N_2_O)
		T3 Cytomel	T3 cytomel	Cytomel (C_15_H_11_I_3_NaO_4_) Triiodothyronine (C_15_H_12_I_3_NO_4_)
Post-cycle therapy		Clomid 50 mg	Clomifene citrate	Clomifene (C_26_H_28_ClNO)
		Nolvadex 20 mg	Tamoxifen citrate	Tamoxifen (C_26_H_29_NO)
		Proviranos 50 mg	Mesterolone	Mesterolone (C_20_H_32_O_2_)
		Proviron 25 mg	Mesterolone	
		Anastrozolos 1 mg	Anastrozol	Anastrozol (C_17_H_19_N_5_)
	Injection	HCG 5000 UI	Pregnyl	Polypeptide
		HCG 1500 UI	Pregnyl	
Other substances		IGF1 Lr3 - Getropin	Insulin-like Growth Factor 1	
		HGH	HGH 100 UI - Getropin	Getropin (C_990_H_153_N_262_O_300_S_7_)
		Eporex 300 (EPO)	Erythropoietin	Erythropoietin (C_809_H_1301_N_229_O_240_S_5_)
Side Effects Medications	Oral	Finasteride - Prostacare	Finasteride	Finasteride (C_23_H_36_N_2_O_2_)
		Viagra	Sildenafil citrate	Sildenafil (C_22_H_30_N_6_O_4_S)
		Cialis	Tadalafil	Tadalafil (C_22_H_19_N_3_O_4_)

Drugs used by AAS consumers are not confined to anabolic steroids. Up to 90 % of AAS users have a palate for polypharmacy, taking a mix of muscle-shaping drugs, in addition to stacking different brands of steroids [6].

These “steroid-accessory” drugs are used for a variety of reasons and can be grouped according to their desired effect (Table 2). Some of these accessory drugs are potentially more dangerous than AAS; the unsupervised use of insulin, diuretics, and thyroxin can precipitate a number of medical emergencies [17].

**Table 2:** Accessory Drugs and Dietary Supplements [17]

Drug/Supplement	Reason for use
Ephedrine	Stimulant, fat loss
Clenbutarol	Stimulant, fat loss
Amphetamine	Stimulant, fat loss
Thyroxine	Thyroid hormone, fat loss
Growth hormone	Anabolic, increase muscle mass and strength
Insulin	Anabolic, increase muscle mass
Insulin-like growth factor	Anabolic, increase muscle mass
Diuretics	Reduce edema
Human chorionic gonadotrophin	Restore endogenous testosterone
Tamoxifen	Prevent gynecomastia
Gamma-hydroxybutyrate	Sedative, aids sleep/releases growth hormone
Opioids	Pain relief
Androstenedione	Over-the-counter testosterone precursor
Creatine	Over-the-counter ergogenic supplement
Dihydroepiandosterone	Over-the-counter steroid precursor

### Physiological and clinical effects

The physiological direct effects of testosterone and AASs (AR-mediated) are well known. They include increases in renal erythropoietin stimulated hæmatopoiesis, lipolysis, protein synthesis, sebaceous secretion, hair growth and libido [13].

Concerning the muscle fiber hypertrophy, a study showed that increases in muscle volume in healthy eugonadal men treated with graded doses of testosterone are associated with concentration-dependent increases in cross-sectional areas of both type I and type II muscle fibers and myonuclear number [18].

However, the indirect effects should also be considered. These include antiglucocorticoid effects, which are mediated by testosterone occupation of cortisol receptors (which have a remarkable affinity with testosterone) and create an anti-catabolic effect [19].

### Side effects

Their incidence is unclear, as the denominator of AAS use is not clear. Acne, alopecia and Lower Urinary Tract Symptoms (LUTS) attributable to prostate enlargement are usually related to the strong androgenic 5DHT-effect. Erectile dysfunction and libido loss may also occur, especially after discontinuation, when endogenous testosterone levels are usually low [13].

A sustained increase in testosterone levels during ‘cycles’ leads to higher aromatization rates of testosterone, which accounts for the gynecomastia typically found in steroid users. Hepatic effects are most often related to oral alkylated agents. They include the uncommon hepatic peliosis, cholestatic jaundice and hepatic neoplasms, such as focal nodular hyperplasia, which are all closely related to dose and duration of usage [20].

Hepatocellular carcinoma and Wilm’s tumour are serious and rare side effects that are always related to long-term and heavy use. Interestingly, there are no recent reports linking AASs to prostate cancer or significant increases in PSA levels. The most severe consequences of long-term AAS use are associated with the cardiovascular system. Hypertension, arrhythmia, erythrocytosis and ventricular dysfunctions have been reported. Mortality risk among chronic users is estimated to be 4.6 times higher than among non-users. Cases of renal failure secondary to rhabdomyolysis and diffuse membranoproliferative glomerulonephritis in heavy users have been reported. Aggressive behaviour, depression, mood swings, altered libido, euphoria and even psychosis are some of the psychiatric patterns related to AAS [21].

Overpharmacy may increase the risk of violent criminality. Withdrawal syndrome and dependency were also described, and the likelihood of psychiatric effects is greater where there is previous psychiatric history, or alcohol or drug abuse [22].

During AAS withdrawal, depressive episodes were reported among untreated AAS users in addition to the significant decrease on the IIEF sexual desire subscale [23].

## Impact on fertility

### Androgen and spermatogenesis

Androgens play a crucial role in the development of male reproductive organs such as the epididymis, vas deferens, seminal vesicles, prostate and penis. In addition, androgens are necessary for puberty, male fertility and male sexual function. High levels of intratesticular testosterone secreted by Leydig cells, are required for spermatogenesis. The AR is in all male reproductive organs and can be stimulated either by testosterone or its potential metabolite: dihydrotestosterone [24].

### Classic reversible AAS-induced hypogonadotropic hypogonadism

Exogenous administration of testosterone synthesis derivatives induces negative feedback on the hypothalamic-pituitary axis and therefore inhibiting the secretion of both FSH and LH. Infertility after AAS abuse commonly presents as oligozoospermia or azoospermia, associated with abnormalities in sperm motility and morphology [25].

### Innovative experimental AAS-induced findings

#### Histopathology

Experiments in animal models mainly report AAS-induced Leydig cell alterations, but cellular morphology anomalies have also been reported [26]. Nethertheless, specific end-stage spermatogenesis impairment, with a lack of advanced forms of spermatids, has been described [27]. After AAS discontinuation, Leydig cells tend to proliferate but remain below the regular counts, even after longer periods [28].

#### Apoptosis

Apoptosis has been reported to play an important role in the regulation of germ cell populations in the adult testis. Recently, the correlation between apoptosis and high AAS doses and exercises has been experimentally assessed in animal models. Shokri et al. report a significant increase in the rate of apoptosis of spermatogenic cells after nandrolone administration, an increase clearly amplified by physical exercise [29].

## Aneuploidies and ultrastructural changes in spermatozoa

The innovative use of both transmission electron microscopy and fluorescence in situ hybridization (FISH) has recently been reported in an AAS user sperm sample, searching for genetic and ultrastructural consequences of steroid abuse. Immaturity, necrosis and apoptosis were assessed, and a high percentage of structurally normal spermatozoa were found, which showed the absence of a correlation between AAS and ultrastructural sperm changes. In contrast to these findings, FISH sperm analysis revealed XY and chromosomes 1 and 9 disomies, suggesting anomalies in the meiotic process and genetic damage among AAS users [30].

### Sperm parameters

The use of a combination of hCG and steroids is a common practice among AAS users. The aim is to avoid the impact of the negative feedback on LH after long term AAS administration, which may lead to a persistent state of hypogonadism and poor sperm quality. In a study of colts, the long-term effects of the anabolic steroid 19-norandrostenololylaurate on sperm characteristics were studied in three experiments [31]. It was concluded that the adverse effects of corticosteroids on semen characteristics were reversible, at least in the treated groups at the age of 7–25 months. In another study on sperm parameters, it was concluded that according to the duration of use of anabolic steroids and the period since the last drug administration prior to the survey, the percentages of motile sperm and whose form is normal were significantly reduced among bodybuilders compared to healthy volunteers [32]. These results suggest that, even after prolonged use of extremely high doses of anabolic steroids, sperm production can return to normal rates for bodybuilders who stopped the consumption of anabolic steroids over 4 months ago.

## Treatment modalities

The infertility treatment with testosterone does not improve spermatogenesis; the administered exogenous testosterone and its metabolite, estrogen, suppresses the production of Gonadotropin-releasing hormone (GnRH) by the hypothalamus and the production of luteinizing hormone (LH) by the pituitary gland, and therefore the production of testicular testosterone.

### Reversible Anabolic-Steroid-induced hypogonadism (ASIH)

According to most reports, the quality of sperm tends to normalize spontaneously within 4–12 months after cessation of anabolic steroid abuse [32]. However, the negative effect on sperm quality may persist for long periods. Simply stopping the use of ASA may lead to the resumption of fertility in a certain proportion of male users [33, 34]. Patients may also be treated by induction of spermatogenesis with gonadotropins or gonadoliberin analogues, including hCG IM injections, human menopausal gonadotropin (hMG) or even recombinant FSH. The use of hCG alone or in combination with hMG was reported as an effective treatment for this group of patients [35]. The restoration of fertility has been reported, even in situations of persistent azoospermia up to 5 years after the AAS stop [32]. Several different regimens are described including testosterone replacement therapy (TRT), Selective Estrogen Receptor Modulator (SERM) such as clomiphene citrate and tamoxifen [36]. For resistant cases, injections of human Chorionic Gonadotropin (hCG) can be used in association with SERM treatment [36].

### Withdrawal treatment

Stopping the use of large doses of anabolic steroids in the long term can lead to the development of withdrawal symptoms. They include: mood disorders (suicidal depression), insomnia, anorexia, decreased libido, fatigue, headache, muscle and joint pain and the desire to take more steroids. Drugs that are targeted to relieve these symptoms include antidepressants, non-steroidal anti-inflammatory and clonidine.

### Difficulties in overall therapeutic care

Comparisons between patients and control case series are difficult because of the concealing of the practice, but also due to various changes in consumption practices and doses employed [37]. However, male infertility related to the abuse of AAS is underdiagnosed and yet it is a potentially curable form. In the absence of spontaneous recovery of spermatogenesis following the cessation of the AAS and after exclusion of other causes of infertility, hormonal therapy should be initiated as a therapeutic alternative [25].

## Conclusion

Anabolic steroids are able to increase strength and muscle mass in some people when combined with a proper diet and an intense training program. All anabolic steroids are also androgenic. Anabolic steroids are marked with numerous side effects, some of which are potentially fatal, and some of which are permanent. However, most side effects are mild and reversible. Education alone is probably not the miracle strategy inciting stopping the abuse of anabolic steroids, but is an essential first step in the fight against this problem. The common factor in the medical literature available on the misuse of AAS is very heterogeneous. This limits objective comparisons between the different drugs and diets. The AAS abuse can disrupt the health of the person at multiple levels. The impact on male fertility is one of the least reported, but certainly one that clinicians should know better. The infertility evaluation of a AAS consumer should include a physical examination, a seminal analysis, a study of hormonal profile and genetic analysis. Immediate cessation of the use of AAS should be encouraged. A lack of awareness regarding the negative long-term effects on fertility was the primary factor related to regret of AAS use in men with anabolic-/steroid-induced hypogonadism [38]. A particular attention should be paid to dietary supplements called “without steroids”. We are lead to the conclusion that the impact of steroids on male fertility is not just a purely transitory state. In short, the best policy is to strongly discourage the use of steroids and, for consumers who persist in their abuse, to offer them an appropriate ethics and clinical uro-andrologic support.

## Abbreviations

5DHT: 5*α*-DiHydroTestosterone
AAS: Anabolic-Androgenic Steroids
AR: androgen receptor
ASIH: Anabolic steroid-induced hypogonadism
FSH: follicle stimulating hormone
GnRH: Gonadotropin-releasing hormone
hCG: human Chorionic Gonadotropin
hMG: human Menopausal Gonadotropin
IIEF: International index of erectile function
IM: intramuscular
LH: luteinizing hormone
LUTS: Lower Urinary Tract Symptoms
PSA: prostate specific antigen
WHO: World Health Organization
